# Health status decline in α-1 antitrypsin deficiency: a feasible outcome for disease modifying therapies?

**DOI:** 10.1186/s12931-018-0844-6

**Published:** 2018-07-20

**Authors:** Robert A. Stockley, Ross G. Edgar, Sian Starkey, Alice M. Turner

**Affiliations:** 1 Lung Investigation Unit, University Hospitals Birmingham NHS Foundation Trust, Queen Elizabeth Hospital Birmingham, Mindelsohn Way, Edgbaston, Birmingham B15 2GW UK; 2 Therapy Services, University Hospitals Birmingham NHS Foundation Trust Queen Elizabeth Hospital Birmingham, Mindelsohn Way, Edgbaston, Birmingham B15 2GW UK; 3 Institute of Applied Health Research, University of Birmingham, Birmingham, B15 2TT UK; 4 Institute of Translational Medicine, University Hospitals Birmingham NHS Foundation Trust Queen Elizabeth Hospital Birmingham, Mindelsohn Way, Edgbaston, Birmingham B15 2GW UK; 5 Heart of England NHS Foundation Trust, Respiratory Medicine, Bordesley Green East, Birmingham, B9 5SS UK

**Keywords:** Alpha-1 antitrypsin deficiency, Health related quality of life, Disease progression

## Abstract

**Background:**

Trials of disease modifying therapies in Chronic Obstructive Pulmonary Disease (COPD) provide challenges for detecting physiological and patient centred outcomes. The purpose of the current study was to monitor decline in health status in Alpha-1 antitrypsin deficiency (AATD) and determine its’ relationship to conventional physiology.

**Methods:**

Patients recruited to the UK-AATD database with a median follow up of 7 years (IQR 5–10) were studied to determine annual change in St George’s Respiratory Questionnaire (SGRQ), FEV_1_, gas transfer and their feasibility of use in future trials.

**Results:**

Annual decline in SGRQ had a wide range, was greater for patients with established COPD and correlated with decline in FEV_1_ (*p* < 0.0001). Total score decline was greater (*p* < 0.05) for those with accelerated FEV_1_ decline (median = 1.07 points/year) compared to those without (median = 0.51). Power calculations indicated effective intervention would not achieve MCID for the SGRQ unless the timeframe was extended for up to 8 years. More than 5000 patients/arm would be required for a statistically significant modest effect over 3 years even in those with rapid FEV_1_ decline.

**Conclusion:**

Despite AATD being a rapidly declining form of COPD, deterioration in SGRQ was slow consistent with ageing and the chronic nature of disease progression. Power calculations indicate the numbers needed to detect a difference with disease modifying therapies would be prohibitive especially in this rare cause of COPD. These data have important implications for future study design of disease modifying therapies even in COPD not associated with AATD.

**Electronic supplementary material:**

The online version of this article (10.1186/s12931-018-0844-6) contains supplementary material, which is available to authorized users.

## Background

Unlike interventions that have short or medium term effects in COPD (improvement in FEV_1_, health status or reduction in exacerbations), therapies that modify disease progression present greater challenges to objective and subjective measures of efficacy. Physiological progression has classically been characterised by FEV_1_ decline although recent COPD studies indicated that not all patients decline [1] leading to attempts to identify “rapid decliners” for study enrichment by patient characteristics such as Emphysema [2] biomarkers such as CC16 [3] or neutrophilic proteinase footprints [4].

Health related outcomes relate to both FEV_1_ [5] and exacerbations [6]. A mean clinically important difference (MCID) is defined for the Saint George’s Respiratory Questionnaire (SGRQ), and is a preferred secondary and co-primary outcome by the US Food and Drug Administration (FDA) recognising its clinical relevance [7, 8]. However entry into clinical trials alone improves both compliance and health status (HS) and clinical impact has to be judged above this placebo effect.

Large COPD trials provide some evidence of disease modification (slowing FEV_1_ decline) in post hoc analysis of TORCH [9] and prospective analysis of SUMMIT studies [10]. However in rarer conditions predisposing to COPD, like Alpha-1 antitrypsin deficiency (AATD), where treatments such as augmentation therapy might be expected to modify disease progression, it is impractical to deliver powered studies with conventional outcomes. The number needed to determine the effect on FEV_1_ decline is deemed prohibitive [11] so a more direct measure of the pathology (emphysema progression) by Computed Tomography has been accepted as a surrogate outcome by the FDA [12]. Nevertheless despite consistent double blind studies of disease modification [13–15] there remains uncertainty about whether augmentation improves other outcomes, such as HS, although there is observational evidence that mortality is reduced [16].

Disease modification in COPD is becoming an important pharmaceutical objective and several anti-inflammatory and anti proteinase strategies are being developed and undergoing phase1/2 testing. The delivery of phase 3 clinical trials needs to address the use of SGRQ in trial design as outlined by the FDA [7, 8]. The purpose of the current study was to determine whether HS defined by the SGRQ could be employed as part of future AATD trials to provide clearer evidence of patient benefit and how the decline of lung physiology influences the SGRQ decline in a cohort of closely monitored patients with AATD never treated with antitrypsin augmentation therapy.

## Methods

The UK AATD registry collects extensive annual demographic data including post bronchodilator lung function (to ERS /ARTP standard) and SGRQ as described [17]. All PiZZ patients (*n* = 454) where SGRQ was available for at least 4 consecutive annual visits, were analysed. The decline in SGRQ total score and the domains were assessed by linear regression analysis to determine change/year. Data was compared between GOLD stages [18] and subgroups defined as non and rapid decliners (≥1% predicted decline per year) for FEV_1_ and Kco as described previously [19]. Lung function was normalised for age, sex, height and ethnicity using the normal ranges described [20]. All patients gave written consent and the study had ethical approval (South Birmingham LREC 3359a).

### Statistical analysis

Statistical analysis used STATA 14 (StataCorp LLC, Texas, USA). A significance level of *p* < 0.05 was used with single tailed tests. Data was not normally distributed, hence is expressed as median and interquartile range. Correlations between baseline and longitudinal SGRQ and pulmonary physiology were determined by linear mixed modelling and differences between groups assessed by Mann-Whitney two sample statistic (two independent variables) and Kruskal Wallis (group wise comparisons). Missing data underwent a case deletion if greater than 5%.

Sample size calculations for a study with SGRQ as the primary outcome together with potential physiological outcomes were based upon a two group parallel comparison, using the formula *n* = 1 + 2*C* (S/D)2 where ***D*** was the smallest difference to be detected (a 25% change in SGRQ decline was used as an arbitrary effect size for illustrative purposes) and ***s*** represented the standard deviation of the observations. ***C*** = 10.51 to provide a 90% power of detecting a change in SGRQ at the 5% level of significance. As the deterioration in HS was determined by slope analysis over at least 4 annual assessments, power would be relevant for a 3 year study.

## Results

### Patient cohort

Data was collected from 454 patients with AATD (PiZZ) with median follow up of 7 years (IQR 5–10). Demographics of patients with and without airflow obstruction (FEV_1_ /FVC ratio greater or less than 0.7) [18] are shown in Table 1. The majority with airflow obstruction were male (64%) and 89% were index cases diagnosed due to respiratory symptoms. The majority were ex smokers (81%):- no current smokers were included in the analysis as there were too few to analyse meaningfully. Patients without COPD were mainly female (61%), non index cases, never smokers and younger (Table 1). There was a wide range of pulmonary function from normal to very severe COPD (GOLD stage 4) also reflected in the gas transfer results and SGRQ scores for the groups with and without COPD.

**Table 1:** Patient characteristics

	No Obstruction			Obstruction			*p*
	N	Median	IQR	N	Median	IQR	
Male n(%)	33 (39%)			235 (64%)			< 0.001
Age	84	42.4	35.5–53.9	370	52.5	46.4–58.5	< 0.001
Index n(%)	39 (46%)			331 (89%)			< 0.001
Never Smoker n(%)	59 (70%)			72 (19%)			< 0.001
Pack Year History	25	6.0	2.0–12.8	296	19.0	10.0–28.0	< 0.001
BMI	84	25.9	23.1–29.9	370	25.1	22.8–27.9	0.043
Baseline							
FEV_1_% predicted	84	113.8	97.9–123.0	370	49.2	36.5–66.1	< 0.001
FVC % predicted	84	115.0	102.2–129.0	370	108.3	94.8–124.3	0.025
FEV_1_ / FVC Ratio	84	80.6	77.2–86.0	370	37.6	30.3–48.0	< 0.001
Tlco % predicted	84	92.5	80.8–108.9	368	66.2	52.5–77.1	< 0.001
Kco % predicted	84	91.1	79.3–100.3	368	63.6	52.1–74.3	< 0.001
SGRQ Symptoms	84	30.9	11.7–57.1	370	62.5	46.2–78.6	< 0.001
SGRQ Activity	84	12.2	0–41.6	370	60.4	47.4–79.7	< 0.001
SGRQ Impacts	84	5.8	0–21.4	370	34.9	21.3–49.9	< 0.001
SGRQ Total	84	14.0	4.8–35.5	370	48.2	33.9–62.4	< 0.001
Annual Decline							
FEV_1_% predicted Slope/yr	84	−0.25	−1.11 – 0.47	370	−1.02	−1.99 – − 0.03	< 0.001
Kco % predicted Slope/yr	84	−0.92	−1.66 – 0.01	364	−1.13	−1.94 – −0.42	0.063
SGRQ Symptoms Slope/yr	84	0.00	−2.45 – 2.02	370	0.21	−2.29 – 2.15	0.291
SGRQ Activity Slope/yr	84	0.00	−0.78 – 1.37	370	1.18	−0.47 – 3.62	< 0.001
SGRQ Impacts Slope/yr	84	0.14	−0.53 – 0.94	370	0.38	−1.07 – 2.18	0.092
SGRQ Total Slope/yr	84	0.21	−0.76 – 1.06	370	0.66	−0.83 – 2.37	0.025

Demographics of the patient group divided into those with and without airflow obstruction (FEV_1_/FVC ratio above and below 0.7). Data is expressed as median and interquartile range. The annual % predicted decline in lung function and St George’s Respiratory Questionnaire domains are also shown

*BMI* Body mass index, *FEV*_*1*_ Forced Expiratory Volume in 1 Second, *FVC* Forced Vital Capacity, *Tlco* Diffusing Capacity of the Lung for carbon monoxide, *Kco* Transfer Coefficient for carbon monoxide, SGRQ St George’s Respiratory Questionnaire

### Relationship between SGRQ and physiological parameters

**Fig. 1:**
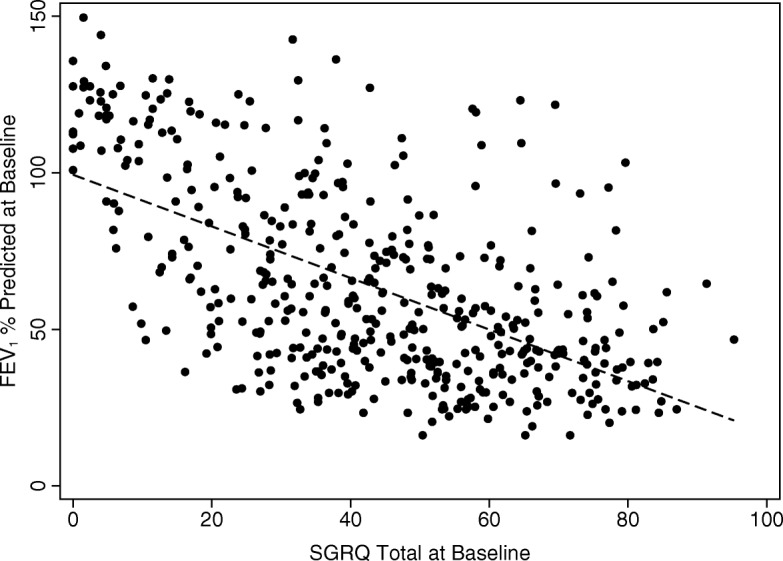
Correlation between FEV_1_ (% predicted) and SGRQ total score at baseline. Each point represents a single patient. The dashed line is a linear regression. *P* < 0.0001 *R*^*2*^ = 0.344

The baseline SGRQ score for the whole group showed a statistically significant (*p* < 0.0001) correlation with baseline FEV_1_ although the r^2^ value of 0.34 indicated that only 34% of the variability in the group was explained by FEV_1_ alone (Fig. 1). The correlations for those with and without COPD were also statistically significant (*p* < 0.0001) although the variance for each group was less (*r*^*2*^ = 0.20 and 0.15 respectively). Similar statistically significant correlations were found between SGRQ total score and gas transfer for the whole group (*r*^*2*^ = 0.105; *p* < 0.0001) and those with COPD (*r*^*2*^ = 0.015; *p* = 0.008) but not those without COPD (*r*^*2*^ = 0.002; *p* = 0.35).

**Fig. 2:**
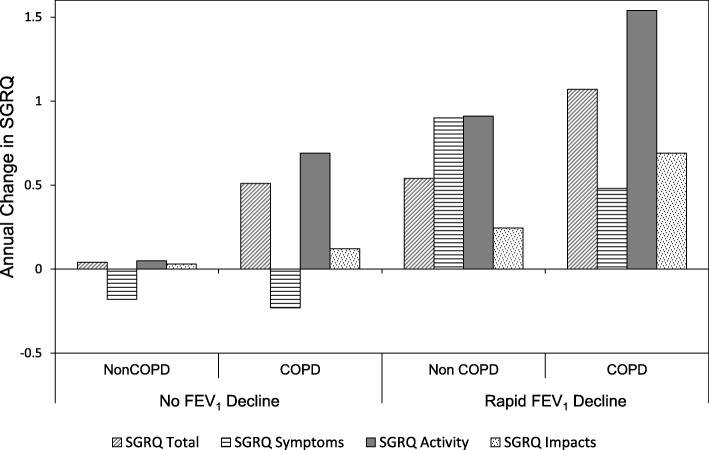
Annual decline in SGRQ total score and its domains. The histograms indicate median values in patients with and without COPD and rapid FEV_1_ decline. Note even those with COPD and rapid FEV_1_ decline have a median total score deterioration well below the MCID

There was a wide range of FEV_1_ and Kco decline (% predicted/year). Generally the SGRQ scores increased yearly for the groups with and without COPD, although again a wide range was seen (Table 1). The symptom and activity score was stable for the non COPD group even though the median total score showed gradual deterioration (+ 0.21 units /year). The group with COPD showed a worsening of symptoms and activity with time and an overall median increase in total score of + 0.66 units/year although only the activity and total score deteriorated more rapidly than for those without COPD (*p* = 0.001 and *p* = 0.025 respectively). Figure 2 shows the annual change in SGRQ and its domains in patients with and without COPD, divided according to no or rapid decline in FEV_1_ [19]. Summary data and demographics of patients in each of these groups is provided in Additional file 1: Tables S1 and S2. In addition the FEV_1_ and Kco decline is summarised in Additional file 1: Tables S3a, b and c for comparison with previous literature that did not account for age sex and height.

**Fig. 3:**
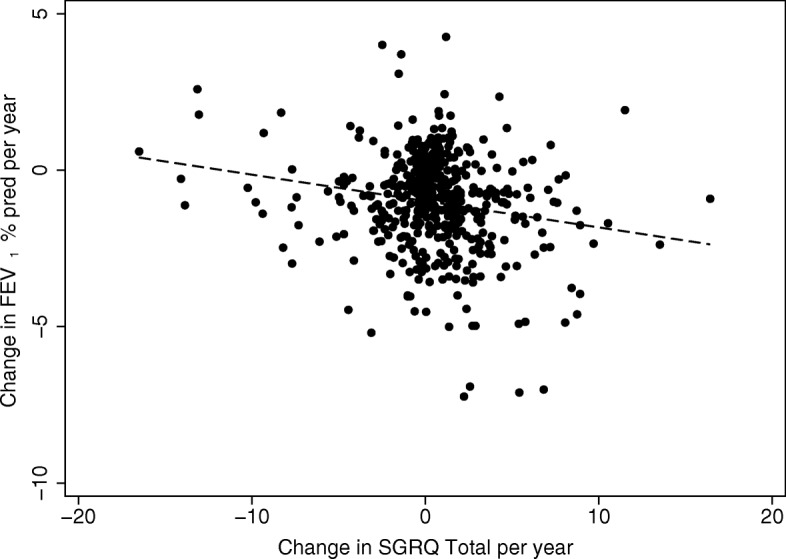
Relationship between deterioration in FEV_1_ and SGRQ. The decline in FEV_1_ is shown compared to the annual change in SGRQ total score. Each point represents data from a single patient. The dashed line is a linear regression, *r*^*2*^ = 0.04, *p* < 0.001

There was no difference in the rate of gas transfer decline between those with and without COPD (*p* = 0.125). There was no relationship between SGRQ decline and gas transfer decline (Additional file 1: Tables S4 and S5). The decline in FEV_1_ was statistically related to the increase in SGRQ total score for the whole group (Fig. 3; *p* < 0.0001), although the relationship (r^2^ 0.04) indicated only 4% of the decline in HS was explained by the decline in FEV_1_ alone. The decline in HS was greater in COPD patient with rapid decline in FEV_1_ (%predicted) compared to those with no decline although only for the total score and activity domain (*p* < 0.05) as shown in supplementary e-Table 2.

**Table 2:** Numbers required to power an augmentation trial

Parameter		No. needed per arm to detect 25% reduction in decline
SGRQ total score	Whole group	8577
	Rapid FEV_1_ decliners	5039
FEV_1_ (% predicted)	Whole group	1516
	Rapid FEV_1_ decliners	86
Kco (% predicted)	Whole group	569
	Rapid Kco decliners	77

The number that is required in each arm with total SGRQ score or lung function as the outcome based on patients with a diagnosis of COPD or only those with known rapid decline in physiology as indicated

*FEV*_*1*_ Forced Expiratory Volume in 1 Second, *Kco* Transfer Coefficient for carbon monoxide, SGRQ St George’s Respiratory Questionnaire

**Fig. 4:**
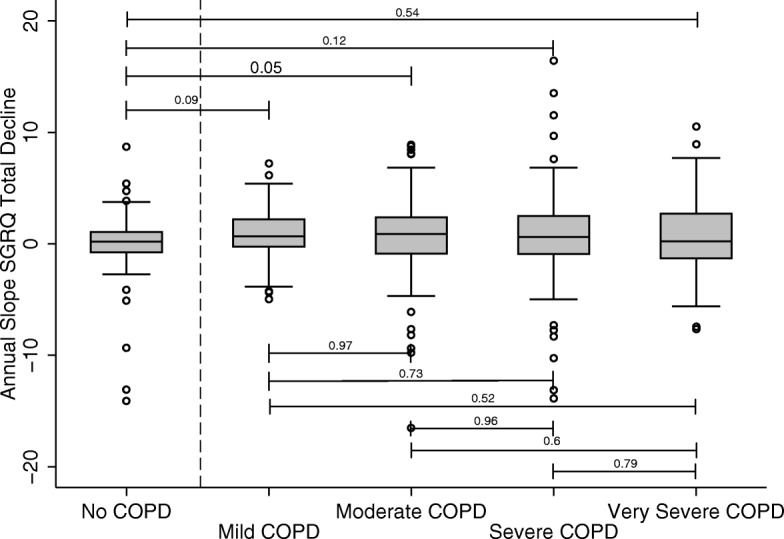
Relationship between GOLD stage and change in SGRQ. The annual change in Total SGRQ is shown for the patient groups separated into the GOLD stages. The data is shown as median with IQR (box), 5–95% range (whiskers) and outliers (open squares). The significance of the difference between groups is shown as the *p* value on the horizontal bars

The decline in SGRQ total score did not differ between GOLD stages (Fig. 4).

### Relationship between change in SGRQ and COPD treatment

Forty eight % of patients without COPD and 2% of those with COPD were not receiving long acting inhaled therapy. The proportion receiving LABA, LAMA or ICS alone were similar for those without and those with COPD (11% vs 19%: 9% vs 9%: and 4% vs 7% respectively). However, only 9% of those without COPD were on triple therapy (LAMA/LABA/ICS) compared to 61% of those with COPD. The high proportion of those without spirometric COPD at the time of referral who were on regular inhaled therapy largely reflects clinical practice at the time of referral and symptoms related to discordance beteen spirometry and gas transfer as described previously [21]. There was no relationship between the decline in HS and the nature of regular inhaled therapy assessed as categorical data.

### Power calculations

Table 2 summarises the numbers needed to confirm a 25% difference in decline in SGRQ total score or FEV_1_ for the group with COPD, and for those with rapid decline in FEV_1,_ assuming 90% power and with a type 1 error set at 5%, The numbers required to detect a 25% difference in Kco decline, in those with COPD and rapid decline is also shown**.**

## Discussion

The SGRQ is a validated, but subjective, measure of a patient’s HS. The current study provides unique and critical data on the effect of AATD on annual HS deterioration over a period of 3 or more years in patients not receiving augmentation therapy, thus reflecting the natural history of the disease. It demonstrates that change in HS relates poorly to physiological progression and that it would not be feasible to establish a trial of disease modifying therapy with SGRQ as a primary outcome. In addition SGRQ is unlikely to provide direct clinical support as a secondary outcome because the numbers required, especially in this rare disease, are prohibitive.

### Patterns of change in health status

Patients were divided into those with and without airflow obstruction and all SGRQ domains and total score showed a wide range of values though significantly worse for those with COPD. However, even in those without COPD the total score and especially the symptom domain score was higher than expected in a healthy population [22]. This likely reflects the proportion of patients in our study who were “index” cases and hence diagnosed after presenting with symptoms. Whereas these patients did not have COPD by spirometric criteria we have shown previously that in AATD the gas transfer can be reduced even in subjects with normal spirometry and likely leads to referral and assessment [21]. This concept is consistent with the lower scores in the non index Swedish birth cohort who have been followed for up to 40 years where HS is maintained and only a small number of current smokers show HS or gas transfer impairment [23].

Changes in HS were greater in those with established COPD, especially the activity score which increased almost + 1.2 units/year, whilst total score change was lower (+ 0.66/year) reflecting the lesser changes in symptom and impact domains. The relationship to GOLD stage indicated those with mild and moderate COPD had the greatest median deterioration in total SGRQ score although there was no statistical difference across groups. Our previous studies had shown little change in average SGRQ scores over 3 years [24] which was at variance with a Dutch AATD study which showed a major decline in total score (+ 6.5 units) over 30 months [25]. The difference likely reflects a combination of the centralisation of patient care and the degree of monitoring carried out at our centre as well as potential improvements in patient care over the intervening 14 years. More recently a study from Germany [26] also documented a slow decline in SGRQ in AATD (+ 1.2 units for total score) which weakly reflected annual exacerbation rates [26]. However, that data was self-reported and included unquantified patients with the SZ phenotype and a significant proportion on augmentation therapy, which may have influenced the results.

The SGRQ domains and total score showed little change in patients with no airflow obstruction. However there was a relationship to the FEV_1_ decline (% predicted): those with rapid decline showed a greater median increase in all domains and total score although the range was wide and only significantly different for the symptom domain. There were differences in the activity domain and total scores when the COPD patients were divided into those with no or rapid decline in FEV_1_. However, when divided into those with rapid or no decline in gas transfer, although the activity domains rose by median values of + 0.18 and + 1.17 units/year respectively, neither achieved statistical significance (Additional file 1: Tables S4 and S5) suggesting the rate of FEV_1_ decline has the more noticeable effect on HS. Although there was an overall (significant) correlation between FEV_1_ decline and SGRQ deterioration, the relationship was weak (Fig. 3).

### Relevance to clinical trial design

These data have important implications for clinical trials of disease modifying therapies in AATD and non deficient COPD. Age itself (without COPD) has an effect on HS [22] although clearly from a lower baseline than in COPD. AATD is considered a more rapidly progressive type of COPD but even those with established COPD stratified for rapid FEV_1_ decline had an overall median annual increase in total SGRQ score that was only + 0.5 points higher than those with no age corrected FEV_1_ decline. Indeed even when stratified into moderate and severe COPD (typical of patients currently included in clinical trials) the differences remained small and statistically insignificant.

Our data was generated from a specialised research programme with annual follow up, which conceivably has a similar placebo effect on HS to that seen in clinical trials [27] and may reflect changes in patient expectations as well as close monitoring and better adherence to background therapy [28]. Despite similar close monitoring, many interventional trials in COPD demonstrate a greater treatment effect on HS parameters (≥ MCID of 4 points for SGRQ total score) which may reflect the nature of the trials aimed at both short term improvement in lung function and modification of exacerbations (e.g. [29, 30]). However, in trials of augmentation therapy in AATD to date no signal has been detected either statistically or close to the MCID [13–15] leading to scepticism about efficacy despite significant effects on emphysema progression [31]. This failure may reflect both the size of the studies in this rare condition and the lack of any immediate improvement in lung function (and hence a noticeable clinical signal) for disease modifying treatments like augmentation.

We observed a slow deterioration in SGRQ scores in patients not receiving augmentation therapy, indicating that detection of slowing of HS decline in clinical trials aimed at disease modification is likely to require very large numbers for adequate power. If augmentation stopped all HS decline it would still require at least a 4 year study in rapid FEV_1_ decliners to achieve the MCID for total SGRQ score. Our data shows that even those with stable FEV_1_ have an annual deterioration in HS which is half that of the rapid FEV_1_ decliners indicating that a study to find a median 4 point effect of treatment that stopped the physiological related decline would take about 8 years.

In the current study we calculated the number needed to confirm a 25% reduction in SGRQ total score decline which, even in pre-identified rapid decliners with COPD (5039 / arm) is prohibitive for a “rare” disease, especially when most identified patients are already on widely accepted AAT augmentation therapy. To detect an SGRQ decline reduced to that seen in COPD patients with a stable age related decline (50% reduction) would still require 1260/arm if treatment were totally effective. Using slope analysis allows projections of the longer term benefit on HS decline, but would still require at least 3 years for decline to be documented and would still not reduce patients required in such a trial to a feasible number.

Slope analysis of health status was preferred to endpoint analysis to overcome any potential regression to the mean resulting in an apparent improvement in scores overtime [32] and at the same time allow more robust longitudinal projection of the changes to determine time to MCID.

Since SGRQ appeared prohibitive as a primary or secondary outcome we also conducted power calculations for a prospective study using both FEV_1_ and Kco as the outcome. Again the numbers appear prohibitive for unselected patients with AATD and COPD. However, the numbers become more feasible if patients were chosen by known rate of rapid physiological decline (Table 2) i.e. enriching the study population for the outcome being assessed. This approach could be considered for both a pragmatic clinical trial and, in the interim, as a criterion for assessing treatment response [19]. Interestingly the power calculations form a rank order consistent with previously published data on the sensitivity of these 3 parameters to change [33].

### Potential strengths and weaknesses

The current study has a potential weakness. It represents a cohort of patients referred to a specialist centre for annual long term follow up as a research programme. Thus it will not represent “usual” clinical care and a degree of selection bias cannot be discounted. However, this is also a strength as the patients and their management likely represent those who would be suitable for, and included in, clinical trials. The results therefore represent what impact a focussed clinical service has on HS and its deterioration and are therefore relevant to the design of trials of disease modifying therapies in AATD where placebo arms should achieve similar results. Equivalent data will also be important in non AATD COPD as use of disease modifying therapies expand to this patient group.

As we were studying HS decline we only included patients in whom this had been documented on at least 4 annual visits. Some patients may not have had this recorded and some may not have returned on at least 4 occasions either because they had become reassured of their physiological “normality” of a combination of distance travelled with and without increased morbidity with time. Nevertheless this selections criterion did not differ with disease severity suggesting it is generally reflective of the patient groups.

The insensitivities of both group physiological data and HS, to change [33] has been an important driving force in the development and validation of CT densitometry as the outcome of choice in emphysema trials especially in AATD. Although lung densitometry relates to both FEV1 and SGRQ cross-sectionally and remains the best predictor of Mortality [34] only the decline in FEV1 has been shown to relate to the decline in lung Density [35]. Whether density decline shows a similar relationship to SGRQ deterioration has not been assessed but such studies may provide indirect evidence of any putative, clinically apparent, benefit that would not be detected by current trial design and power.

## Conclusion

Although HS relates to baseline lung physiology and its decline, the changes are minimal with time even in those with documented rapid physiological decline. These data support the role of close patient supervision and monitoring in a specialist clinic and provides fuel for debate about trial designs to assess therapeutic efficacy of COPD disease modification with HS as an outcome.

## Additional file

Additional file 1: **Table S1.** Demographics of patients without COPD. **Table S2.** Demographics of patients with COPD. **Tables S3.** a-c FEV1 and Kco decline including annual change in absolute units. **Table S4.** SGRQ domains and total scores for non COPD cohort split by those with normal age related decline in Kco and those with rapid decline. **Table S5.** SGRQ deterioration for COPD cohort split by normal age related Kco decline and rapid Kco decline. (DOCX 35 kb)

## Abbreviations

AAT: Alpha-1 Antitrypsin
AATD: Alpha-1 Antitrypsin Deficiency
ARTP: Association for Respiratory Technology and Physiology
BMI: Body Mass Index
COPD: Chronic Obstructive Pulmonary Disease
ERS: European Respiratory Society
FDA: Food and Drug Administration
FEV_1_: Forced Expiratory Volume in 1 Second
FEV_1_/FVC: The Ratio of FEV_1_ and FVC
FVC: Forced Vital Capacity
GOLD: Global Initiative for Chronic Obstructive Lung Disease
HS: Health Status
ICS: Inhaled corticosteroid
IQR: Interquartile Range
Kco: Transfer Coefficient for carbon monoxide
LABA: Long acting Beta 2 antagonist
LAMA: Long acting muscarinic antagonist
LREC: Local Research Ethics Committee
MCID: Minimum Clinical Important Difference
n: Number
NIHR: National Institute for Health Research
PkYrHx: Pack Year History
QoL: Quality of Life
SGRQ: St George’s Respiratory Questionnaire
TLco: Diffusing Capacity of the Lung for carbon monoxide
UK: United Kingdom
