# Supplementary material for: Health status decline in α-1 antitrypsin deficiency: a feasible outcome for disease modifying therapies?
Source: Respir Res. 2018 Jul 20;19:137. doi: 10.1186/s12931-018-0844-6 (PMC6053712; doi:10.1186/s12931-018-0844-6)
Supplement: Supplementary file 1 — Table S1. Demographics of patients without COPD. Table S2. Demographics of patients with COPD. Tables S3. a-c FEV1 and Kco decline including annual change in absolute units. Table S4. SGRQ domains and total scores for non COPD cohort split by those with normal age related decline in Kco and those with rapid decline. Table S5. SGRQ deterioration for COPD cohort split by normal age related Kco decline and rapid Kco decline. (DOCX 35 kb) [file 12931_2018_844_MOESM1_ESM.docx]

Additional file 1

|  | | No FEV_1_ Decline | | | FEV_1_ Decline | | | P |
| --- | --- | --- | --- | --- | --- | --- | --- | --- |
|  |  | N | Median | IQR | N | Median | IQR |  |
| Male n(%) | | 10 (29%) | | | 9 (41%) | | | 0.168 |
| Age | | 35 | 39.0 | 30.6 - 52.8 | 22 | 48.1 | 38.1 – 60.5 | 0.037 |
| Index n(%) | | 17 (49%) | | | 10 (45%) | | | 0.409 |
| Never Smoker n(%) | | 23 (66%) | | | 17 (66%) | | | 0.177 |
| Pack Year History | | 12 | 7.8 | 2.6 – 15.4 | 5 | 1.0 | 0.2 – 5.0 | 0.026 |
| BMI | | 35 | 26.9 | 22.9 – 32.2 | 22 | 25.5 | 22.0 – 29.4 | 0.238 |
| Baseline | FEV_1_ % predicted | 35 | 108.9 | 97.9 – 123.0 | 22 | 119.6 | 36.5 – 66.1 | 0.034 |
|  | FVC % predicted | 35 | 104.1 | 102.2 – 129.0 | 22 | 125.0 | 94.8 – 124.3 | 0.002 |
|  | FEV_1_ / FVC Ratio | 35 | 85.0 | 77.2 – 86.0 | 22 | 78.6 | 30.3 – 48.0 | 0.003 |
|  | Tlco % predicted | 35 | 88.5 | 80.8 – 108.9 | 22 | 87.3 | 52.5 – 77.1 | 0.140 |
|  | Kco % predicted | 35 | 94.0 | 79.3 – 100.3 | 22 | 84.5 | 52.1 – 74.3 | 0.009 |
|  | SGRQ Symptoms | 35 | 35.0 | 11.7 – 57.1 | 22 | 30.5 | 46.2 – 78.6 | 0.406 |
|  | SGRQ Activity | 35 | 23.3 | 0 – 41.6 | 22 | 11.7 | 47.4 – 79.7 | 0.250 |
|  | SGRQ Impacts | 35 | 11.2 | 0 – 21.4 | 22 | 5.3 | 21.3 – 49.9 | 0.367 |
|  | SGRQ Total | 35 | 16.2 | 4.8 – 35.5 | 22 | 11.5 | 33.9 – 62.4 | 0.412 |
| Annual Decline | FEV_1_ % predicted Slope/yr | 35 | 0.51 | 0.32 – 0.9 | 22 | -1.94 | -4.00 – -1.03 | <0.001 |
|  | Kco % predicted Slope/yr | 35 | -1.11 | -1.63 – 0.02 | 22 | -1.04 | -1.54 – -0.01 | 0.500 |
|  | SGRQ Symptoms Slope/yr | 35 | -0.18 | -3.04 – 1.55 | 22 | 0.90 | -1.54 – 2.30 | 0.052 |
|  | SGRQ Activity Slope/yr | 35 | 0.05 | -1.37 – 1.34 | 22 | 0.91 | 0.00 – 3.06 | 0.088 |
|  | SGRQ Impacts Slope/yr | 35 | 0.03 | -0.56 – 0.65 | 22 | 0.25 | -1.00 – 1.34 | 0.256 |
|  | SGRQ Total Slope/yr | 35 | 0.04 | -0.72 – 0.75 | 22 | 0.54 | -1.01 – 1.88 | 0.119 |

Table S1: Demographics of patients without COPD

The data is shown divided into those with no decline in FEV_1_ greater than that expected for age and those with a decline of 1% predicted (or more)/ year(rapid decliners). Data is median and IQR.

Legend

BMI=Body mass index, FEV_1_= Forced Expiratory Volume in 1 Second, FVC= Forced Vital Capacity, Tlco= Diffusing Capacity of the Lung for carbon monoxide, Kco= Transfer Coefficient for carbon monoxide, SGRQ= St Georges’ Respiratory Questionnaire, IQR= Interquartile Range.

|  | | No FEV_1_ Decline | | | FEV_1_ Decline | | | P |
| --- | --- | --- | --- | --- | --- | --- | --- | --- |
|  |  | N | Median | IQR | N | Median | IQR |  |
| Male n(%) | | 36 (50%) | | | 124 (66%) | | | 0.010 |
| Age | | 72 | 42.4 | 35.5 - 53.9 | 189 | 52.5 | 46.4 – 58.5 | 0.348 |
| Index n(%) | | 65 (90%) | | | 165 (87%) | | | 0.253 |
| Never Smoker n(%) | | 13 (18%) | | | 45 (24%) | | | 0.003 |
| Pack Year History | | 59 | 18.0 | 11.3 – 27.0 | 142 | 18.0 | 10.0 – 25.0 | 0.180 |
| BMI | | 72 | 25.8 | 24.0 – 28.9 | 189 | 24.4 | 22.2 – 27.2 | 0.001 |
| Baseline | FEV_1_ % predicted | 72 | 48.1 | 30.8 – 62.3 | 189 | 55.8 | 41.9 – 72.1 | 0.006 |
|  | FVC % predicted | 72 | 106.4 | 86.0 – 120.9 | 189 | 113.8 | 96.4 – 129.3 | 0.018 |
|  | FEV_1_ / FVC Ratio | 72 | 37.7 | 27.6 – 50.0 | 189 | 39.7 | 32.5 – 49.5 | 0.073 |
|  | Tlco % predicted | 70 | 70.6 | 52.2 – 83.5 | 189 | 63.8 | 54.1 – 73.9 | 0.078 |
|  | Kco % predicted | 70 | 66.8 | 53.9 – 80.8 | 189 | 61.9 | 50.3 – 70.1 | 0.015 |
|  | SGRQ Symptoms | 72 | 62.4 | 51.5 – 78.1 | 189 | 60.5 | 42.9 – 74.6 | 0.105 |
|  | SGRQ Activity | 72 | 66.6 | 47.7 – 80.9 | 189 | 59.5 | 41.4 – 79.9 | 0.032 |
|  | SGRQ Impacts | 72 | 35.6 | 22.3 – 49.5 | 189 | 34.1 | 17.0 – 47.2 | 0.131 |
|  | SGRQ Total | 72 | 51.8 | 35.0 – 63.3 | 189 | 45.2 | 30.5 – 61.5 | 0.051 |
| Annual Decline | FEV_1_ % predicted Slope/yr | 72 | 0.62 | 0.26 – 1.07 | 189 | -1.95 | -2.79 – -1.41 | <0.001 |
|  | Kco % predicted Slope/yr | 70 | -1.01 | -1.82 – -0.31 | 185 | -1.02 | -1.95– -0.29 | 0.403 |
|  | SGRQ Symptoms Slope/yr | 72 | -0.23 | -1.90 – 1.28 | 189 | 0.48 | -2.64 – 2.65 | 0.117 |
|  | SGRQ Activity Slope/yr | 72 | 0.69 | -0.35 – 2.27 | 189 | 1.54 | -0.47 – 4.3 | 0.045 |
|  | SGRQ Impacts Slope/yr | 72 | 0.12 | -0.91 – 1.45 | 189 | 0.69 | -1.04 – 2.95 | 0.068 |
|  | SGRQ Total Slope/yr | 72 | 0.51 | -0.77 – 1.45 | 189 | 1.07 | -1.07 – 2.89 | 0.047 |

Table S2: Demographics of patients with COPD

Baseline data is given for the patients with COPD divided into those with normal age related decline in FEV_1_ and those with a rapid decline (>1% predicted/year) _1_. The number of patients with data in each group (N) is shown. Data is median and IQR.

Legend

BMI=Body mass index, FEV_1_= Forced Expiratory Volume in 1 Second, FVC= Forced Vital Capacity, Tlco= Diffusing Capacity of the Lung for carbon monoxide, Kco= Transfer Coefficient for carbon monoxide, SGRQ= St Georges’ Respiratory Questionnaire, IQR= Interquartile Range.

tables S3 a-c: FEV_1_and Kco decline including annual change in absolute units.

Data is median and IQR. The p values (single tailed) are shown for the difference between the 2 groups.

Legend: FEV_1_= Forced Expiratory Volume in 1 Second, mls/yr= millilitres per year, mmol/min/kPa/L/yr= millimoles per minute per kilopascal per year, Kco= Transfer Coefficient for carbon monoxide, IQR= Interquartile Range.

|  | **No Obstruction** | | | **Obstruction** | | | P |
| --- | --- | --- | --- | --- | --- | --- | --- |
|  | N | Median | IQR | N | Median | IQR |  |
| FEV_1_ % predicted Slope/yr | 84 | -0.25 | -1.11 – -0.47 | 370 | -1.02 | -1.990 – -0.30 | <0.001 |
| FEV_1_ mls/yr | 84 | -34.95 | -58.62 – -15.23 | 370 | -43.97 | -74.23 – -20.16 | 0.066 |
| Kco % predicted Slope/yr | 84 | -0.92 | -1.66 – 0.01 | 364 | -1.13 | -1.94 – -0.42 | 0.063 |
| Kco % mmol/min/kPa/L/yr | 84 | -0.02 | -0.04 – -0.01 | 364 | -0.02 | -0.04 – -0.01 | 0.369 |

Table S3a: FEV_1_and Kco decline in patients with and without COPD.

The data is shown divided into those with and without airflow obstruction (FEV_1_/FVC ratio above and below 0.7).

|  | **No FEV_1_ Decline** | | | **FEV_1_ Decline** | | | P |
| --- | --- | --- | --- | --- | --- | --- | --- |
|  | N | Median | IQR | N | Median | IQR |  |
| FEV_1_ % predicted Slope/yr | 35 | 0.51 | 0.32 – 0.9 | 22 | -1.94 | -4.00 – -1.03 | <0.001 |
| FEV_1_ mls/yr | 35 | -9.46 | -18.14 – 0.66 | 22 | -101.52 | -126.50 – -77.82 | <0.001 |
| Kco % predicted Slope/yr | 35 | -1.11 | -1.63 – 0.02 | 22 | -1.04 | -1.54 – -0.01 | 0.500 |
| Kco % mmol/min/kPa/L/yr | 35 | -0.03 | -0.04 – -0.01 | 2 | -0.02 | -0.04 – -0.01 | 0.447 |

Table S3b: FEV_1_and Kco decline in patients without COPD The data is shown divided into those with no decline in FEV_1_ greater than expected for age and those with a decline of 1% predicted (or more)/ year(rapid decliners).

|  | **No FEV_1_ Decline** | | | **FEV_1_ Decline** | | | P |
| --- | --- | --- | --- | --- | --- | --- | --- |
|  | N | Median | IQR | N | Median | IQR |  |
| FEV_1_ % predicted Slope/yr | 72 | 0.62 | 0.25 – 1.07 | 189 | -1.95 | -2.79 – -1.41 | <0.001 |
| FEV_1_ mls/yr | 72 | 4.08 | -6.73 – 19.84 | 189 | -73.51 | -98.06 – -58.03 | <0.001 |
| Kco % predicted Slope/yr | 70 | -1.00 | -1.82 – -0.31 | 185 | -1.02 | -1.95 – -0.29 | 0.403 |
| Kco % mmol/min/kPa/L/yr | 70 | -0.02 | -0.03 – -0.01 | 185 | -0.02 | -0.04 – -0.01 | 0.377 |

Table S3c: FEV_1_and Kco decline in patients with COPD.

The data is shown divided into those with no decline in FEV_1_ greater than that expected for age and those with a decline of 1% predicted (or more)/ year(rapid decliners).

| Kco % predicted decline group | statistic | SGRQ | | | |
| --- | --- | --- | --- | --- | --- |
|  |  | Symptoms | Activity | Impacts | Total |
| No Decline | N | 21 | 21 | 21 | 21 |
|  | Median | 0.60 | 0.00 | 0.08 | 0.24 |
|  | IQR | -3.04 – 2.17 | -1.66 – 0.79 | -0.49 – 1.00 | -0.84 – 0.75 |
| Rapid Decline | N | 38 | 38 | 38 | 38 |
|  | Median | -0.30 | 0.26 | 0.28 | 0.21 |
|  | IQR | -2.67 – 1.42 | -1.18 – 2.23 | -0.48 – 1.33 | -0.76 – 0.88 |
| p | | 0.258 | 0.108 | 0.287 | 0.388 |

Table S4: SGRQ domains and total scores for non COPD cohort split by those with normal age related decline in Kco and those with rapid decline

The lack of significant differences between the two groups is confirmed by the p values (single tailed)

Legend

Kco= Transfer Coefficient for carbon monoxide, SGRQ= St Georges’ Respiratory Questionnaire, IQR= Interquartile Range.

| Kco % predicted decline group | statistic | SGRQ | | | |
| --- | --- | --- | --- | --- | --- |
|  |  | Symptoms | Activity | Impacts | Total |
| No Decline | N | 51 | 51 | 51 | 51 |
|  | Median | 0.43 | 0.18 | 0.43 | 0.61 |
|  | IQR | -2.09 – 1.96 | -1.51 – 4.12 | -1.28 – 2.38 | -1.30 – 2.71 |
| Rapid Decline | N | 200 | 200 | 200 | 200 |
|  | Median | 0.12 | 1.17 | 0.22 | 0.54 |
|  | IQR | -2.31 – 2.52 | -0.25 – 3.62 | -1.03 – 2.18 | -0.75 – 2.26 |
| p | | 0.446 | 0.263 | 0.387 | 0.469 |

Table S5: SGRQ deterioration for COPD cohort split by normal age related Kco decline and rapid Kco decline.

The p values (single tailed) are shown for the difference between the 2 groups.

Legend

Kco= Transfer Coefficient for carbon monoxide, SGRQ= St Georges’ Respiratory Questionnaire, IQR= Interquartile Range.
